# HIV-1 Tat Stimulates Transcription Complex Assembly through Recruitment of TBP in the Absence of TAFs

**DOI:** 10.1371/journal.pbio.0030044

**Published:** 2005-02-08

**Authors:** Tamal Raha, S. W. Grace Cheng, Michael R Green

**Affiliations:** **1**Howard Hughes Medical Institute, Programs in Gene Function and Expression and Molecular MedicineUniversity of Massachusetts Medical School, Worcester, MassachusettsUnited States of America; University of California, San DiegoUnited States of America

## Abstract

The human immunodeficiency virus type I (HIV-1) transactivator protein Tat is an unusual transcriptional activator that is thought to act solely by promoting RNA polymerase II processivity. Here we study the mechanism of Tat action by analyzing transcription complex (TC) assembly in vivo using chromatin immunoprecipitation assays. We find, unexpectedly, that like typical activators Tat dramatically stimulates TC assembly. Surprisingly, however, the TC formed on the HIV-1 long terminal repeat is atypical and contains TATA-box-binding protein (TBP) but not TBP-associated factors (TAFs). Tat function involves direct interaction with the cellular cofactor positive transcription elongation factor b (P-TEFb). Artificial tethering of P-TEFb subunits to HIV-1 promoter DNA or nascent RNA indicates that P-TEFb is responsible for directing assembly of a TC containing TBP but not TAFs. On the basis of this finding, we identify P-TEFb-dependent cellular promoters that also recruit TBP in the absence of TAFs. Thus, in mammalian cells transcription of protein-coding genes involves alternative TCs that differ by the presence or absence of TAFs.

## Abstract 2

The transcriptional activator, HIV-1 Tat, not only acts by promoting RNA polymerase processivity, but it is able to promote transcription complex assembly in the absence of TATA- box-binding protein-associated factors

## Introduction

In eukaryotes, gene regulation is largely controlled at the transcriptional level. Factors involved in the accurate transcription of eukaryotic structural genes by RNA polymerase II (class II genes) can be classified into two groups. First, general (or basic) transcription factors (GTFs) are necessary and can be sufficient for accurate transcription initiation in vitro (for review, see [1]). Such factors include RNA polymerase II itself and at least six GTFs: TFIID, TFIIA, TFIIB, TFIIE, TFIIF, and TFIIH. The GTFs assemble on the core promoter in an ordered fashion to form a transcription pre-initiation complex (PIC). The first step in PIC assembly is binding of the GTF TFIID to the TATA box. TFIID is a multi-subunit complex consisting of the TATA-box-binding protein (TBP) and a set of tightly bound TBP-associated factors (TAFs) [2,3,4].

Transcriptional activity is greatly stimulated by the second class of factors, promoter-specific activator proteins (activators). In general, cellular activators are sequence-specific DNA-binding proteins whose recognition sites are usually present in sequences upstream of the core promoter (reviewed in [5,6]). In addition to a sequence-specific DNA-binding domain, a typical activator also contains a separable activation domain. A variety of studies indicate that activators work, at least in part, by increasing PIC formation through a mechanism thought to involve direct interactions with one or more components of the transcription machinery [1,7,8,9]. Activators can also act through other mechanisms, such as increasing the rate of transcriptional elongation, promoting multiple rounds of transcription, and directing chromatin modifications (reviewed in [10]).

The Tat protein of human immunodeficiency virus type I (HIV-1) is a potent activator of the viral long terminal repeat (LTR) and is required for viral replication (reviewed in [11,12,13]). Unlike typical activators, which bind to promoter DNA, Tat binds to nascent viral RNA through an RNA-binding site termed the TAR element. Tat function involves direct interaction with the cellular cofactor called positive transcription elongation factor b (P-TEFb), which is composed of two subunits, cyclin T1 (CycT1) and CDK9 (reviewed in [14,15]). Several lines of evidence have suggested that unlike typical activators, Tat stimulates transcriptional elongation rather than initiation. According to the current model (reviewed in [11,12,13]), in the absence of Tat, RNA polymerase II initiates from the HIV-1 LTR in a form unable to elongate efficiently and thus stalls (or pauses) near the transcription start site. When present, Tat binds to TAR and recruits P-TEFb, allowing CDK9 to phosphorylate the C-terminal domain of the RNA polymerase II large subunit, thereby increasing transcriptional processivity.

The chromatin immunoprecipitation (ChIP) assay has provided a powerful approach to study in vivo the mechanism by which activators stimulate transcription and to delineate the composition of transcription complexes (TCs). In yeast, ChIP analysis has been used to show that activators function, at least in part, by stimulating TC assembly. These studies have also revealed that at certain promoters TBP is recruited in the absence of TAFs [16,17], indicating that, at least in yeast, some TCs contain TBP but not TFIID. Here we perform ChIP experiments to study the mechanism of transcription activation by Tat in vivo.

## Results

### Experimental Design

To analyze transcription stimulation by Tat and other activators we performed ChIP experiments in transiently transfected mammalian tissue culture cells. Plasmids expressing a reporter construct, containing a core promoter and various combinations of activator-binding sites, were transiently transfected into HeLa or 293T cells. In some experiments, a second plasmid expressing an activator was co-transfected. Reporter activity or primer extension was used to quantitate transcription levels. TC assembly was analyzed by a ChIP assay using antibodies directed against components of four representative GTFs involved in distinct stages of TC assembly: TFIID (TBP and typically TAF1 and TAF5), TFIIB, mediator (CDK8 and hMed6), and RNA polymerase II (RBP1). In most experiments the analysis was performed using two sets of primers: one set encompassed the core promoter, transcription start site, and immediate downstream region, and a second set was located far downstream of the transcription start site, within the open reading frame (ORF).

### Diverse Activators Function by Increasing TC Assembly

In yeast, it has been shown that activators stimulate TC assembly, which is evident at the earliest step of this process, interaction of TBP with the TATA box [18,19]. To confirm that this was also the case in mammalian cells, we used a ChIP assay to analyze TC assembly in three well-characterized model systems: transcription directed by Gal4-VP16, transcription directed by the SV40 enhancer, and transcription directed by the adenovirus (Ad) E1a protein.

**Figure 1:**
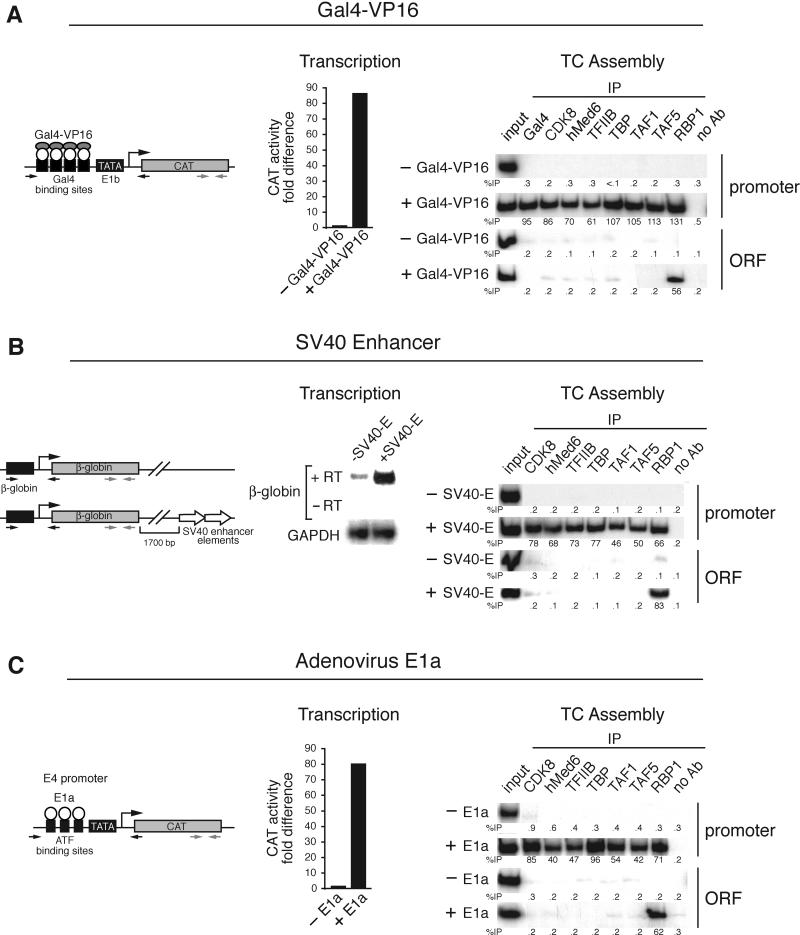
Diverse Activators Function by Increasing TC Assembly (A) Gal4-VP16. Left: a CAT reporter construct containing the E1b core promoter and four Gal4-binding sites (G4E1bCAT) was transiently trans-fected into 293T cells, together with a plasmid expressing Gal4-VP16. Middle: transcription levels were determined by quantitating CAT reporter activity. Right: TC assembly was analyzed by a ChIP assay using the indicated antibodies. The percentage of DNA immunoprecipitated relative to input is indicated. The location of the primers used in the ChIP assay to analyze the promoter (black) or ORF (gray) is schematically shown on the left. (B) SV40 enhancer. Shown on the left is a schematic diagram of a construct containing a minimal rabbit *β*-globin promoter and harboring or lacking the SV40 enhancer. Also shown is transcription analysis by CAT assay (middle) and TC assembly (right) in transiently transfected HeLa cells. (C) Ad E1a. Left: a CAT reporter construct containing the Ad E4 promoter and upstream ATF-binding sites (E4CAT) was transiently transfected into HeLa cells, together with a plasmid expressing Ad E1a. Middle: transcription analysis by CAT assay is shown. Right: TC assembly is shown.

The artificial activator Gal4-VP16 contains an unusually potent acidic activation domain and can stimulate transcription in many species and cell types including mammalian tissue culture cells [5]. Figure 1A shows, consistent with previous studies, that on a synthetic promoter containing four Gal4-binding sites upstream of the TATA box, transcription was not detectable in the absence of Gal4-VP16, whereas addition of Gal4-VP16 led to a large transcriptional increase. The accompanying ChIP assay shows that in the absence of Gal4-VP16 there was no detectable association of GTFs with the promoter. However, addition of Gal4-VP16 led to a large increase in recruitment of all GTFs analyzed, explaining the transcriptional stimulation. Consistent with the transcription results, RNA polymerase II was associated with the ORF in the presence but not absence of Gal4-VP16. Also, as expected, the GTFs were associated with the core promoter and not the ORF.

Transcription of certain genes requires a *cis-*acting enhancer element that is often located at a distance from the transcription start site (reviewed in [20]). The enhancer functions by providing binding sites for cellular activators. For example, a minimal *β-globin* promoter is inefficiently expressed, but addition of an appropriate enhancer element dramatically increases transcription [21]. Figure 1B shows, as expected, that a minimal rabbit *β-globin* promoter was virtually inactive, whereas transcriptional activity was greatly increased by addition of the SV40 enhancer. The ChIP analysis shows that in the absence of the SV40 enhancer there was no detectable association of GTFs with the *β-globin* promoter. Upon addition of the SV40 enhancer, there was a large increase in GTF recruitment that correlated with the increased transcriptional activity.

Finally, we analyzed transcription directed by the Ad E1a protein. Efficient transcription from Ad early promoters requires the viral E1a protein as well as the participation of cellular transcription factors that also bind to the promoter (reviewed in [22]). For example, the Ad E4 promoter contains multiple binding sites for cellular ATF proteins, which are required for efficient transcription activation by E1a [23]. Figure 1C shows, as expected, that in the absence of E1a there was only a background level of Ad E4 transcription, and that E1a increased transcription dramatically. The accompanying ChIP assay shows that in the absence of E1a there was no significant association of GTFs with the promoter, whereas transfection of E1a resulted in recruitment of all GTFs analyzed in a manner that paralleled the increased transcriptional activity.

In summary, the results of Figure 1 indicate, as in yeast, that in three well-studied higher eukaryotic examples, transcription activation involves promotion of TC assembly. Moreover, as in yeast, the stimulatory effect is evident at the earliest step of TC assembly, the TBP– TATA box interaction.

### The HIV-1 Tat Protein Stimulates TC Assembly through Recruitment of TBP in the Absence of TAFs

**Figure 2:**
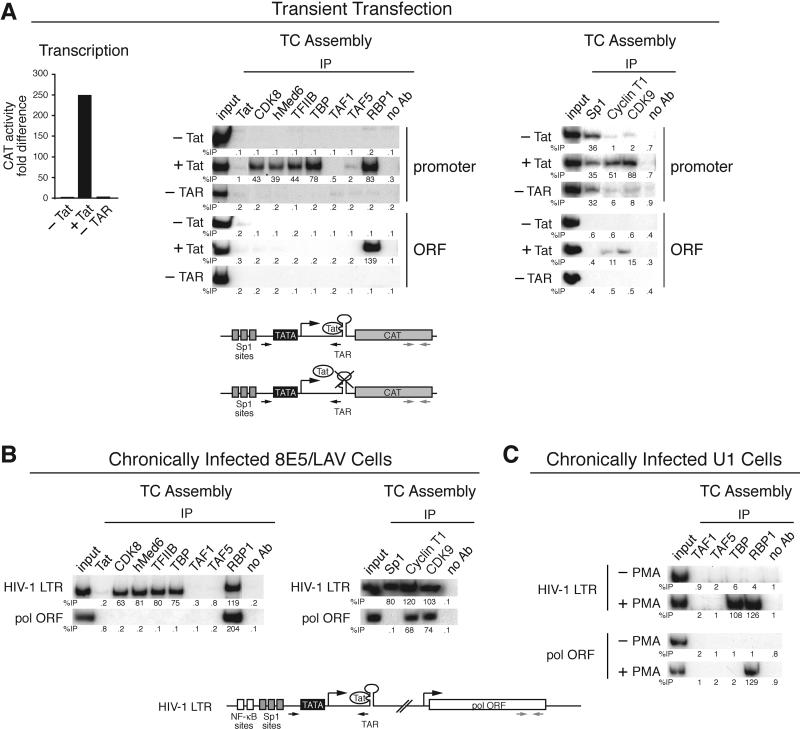
The HIV-1 Tat Protein Stimulates TC Assembly through Recruitment of TBP in the Absence of TAFs (A) A CAT reporter construct containing the TAR element and Sp1-binding sites ([−83]HIV LTRCAT) was transiently transfected into 293T cells, together with a plasmid expressing Tat. Left: transcription analysis by CAT assay is shown. Middle and right: TC assembly is shown. Bottom: a schematic diagram of the CAT reporter construct is shown. (B) TC assembly was monitored in a chronically HIV-1-infected cell line, 8E5/LAV, that harbors an integrated provirus that is constitutively transcribed. Virus production was confirmed by analysis of p24 levels and reverse transcriptase activity in the culture medium (data not shown). (C) TC assembly was monitored in the chronically infected cell line U1 on the integrated HIV-1 LTR in the presence or absence of PMA.

We next investigated Tat-mediated transcription activation and TC assembly on the HIV-1 LTR promoter. Figure 2A shows, as expected, that in the absence of Tat there was no detectable transcription from the HIV-1 LTR, and that addition of Tat increased transcription dramatically. The accompanying ChIP experiment (Figure 2A, left) shows that in the absence of Tat, association of GTFs with the core promoter was virtually undetectable. Sp1, a constitutive cellular activator that binds upstream of the HIV-1 core promoter was, as expected, associated with the promoter in the absence of Tat and unaffected by Tat addition (Figure 2A, right). Significantly, in the absence of Tat there was no detectable association of RNA polymerase II near the transcription start site or within the ORF (Figure 2A, left). However, following addition of Tat, there was a large increase in association of TBP, TFIIB, mediator, and RNA polymerase II with the promoter, which paralleled the transcriptional increase. Also, as expected, there was a large increase in association of RNA polymerase II with the ORF. Following recruitment of P-TEFb to the HIV-1 LTR by Tat, P-TEFb associates with and phosphorylates RNA polymerase II [12,24]. Accordingly, the ChIP assay (Figure 2A, right) shows that in the presence of Tat the two P-TEFb subunits, CycT1 and CDK9, were present at both the promoter and the ORF. Unexpectedly, although TBP and the other GTFs were efficiently recruited to the promoter in the presence of Tat, there was no significant recruitment of the two TAFs analyzed, TAF1 or TAF5 (left panel).

We note that recruitment of Tat was not observed in the ChIP assay, which detects proteins bound either directly or indirectly to DNA (reviewed in [25]). Unlike all the other transcription factors, which associate with the TC through DNA–protein or protein–protein interactions, Tat is bound to nascent RNA [11,12,13].

To determine whether the lack of TAF recruitment was a general feature of Tat-directed transcription, we analyzed a chronically HIV-1-infected cell line, 8E5/LAV, which harbors an integrated provirus that is constitutively transcribed [26]. Figure 2B shows that TBP, TFIIB, mediator, Sp1, P-TEFb, and RNA polymerase II, but not TAF1 or TAF5, were associated with the integrated proviral promoter, consistent with the results of the transient transfection assay.

We also analyzed a second chronically HIV-1-infected cell line, U1, which has been used as a model to study viral latency. U1 cells harbor an integrated viral genome, but, unlike 8E5/LAV cells, the constitutive level of viral expression is extremely low. Viral expression can be induced by phorbol esters, which stimulate transcription through upstream NF-κB-binding sites in the HIV-1 LTR, leading to Tat synthesis and a subsequent substantial Tat-mediated transcriptional increase [27,28]. Thus, U1 cells provide an experimental system to analyze TC assembly from an integrated HIV-1 LTR in the inactive (−PMA) or active (+PMA) state. Figure 2C shows that following activation of the HIV-1 LTR by PMA addition, there was a large increase in recruitment of TBP and RNA polymerase II, whereas TAF1 and TAF5 were once again not detected.

**Figure 3:**
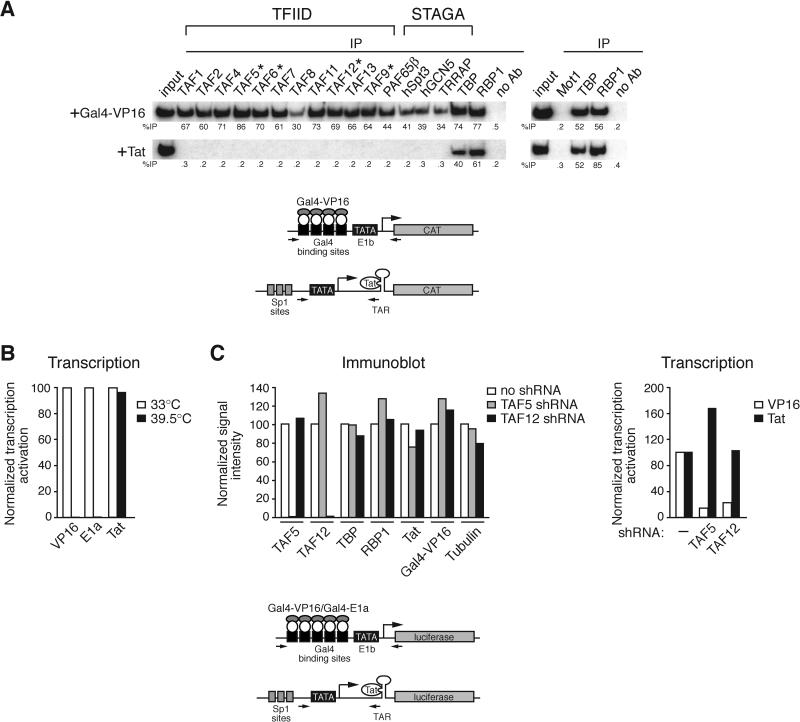
TAFs Are Not Recruited to the HIV 1-LTR and Not Required by Tat for Transcription Activation (A) TC assembly was analyzed by a ChIP assay using antibodies directed against nine additional human TAFs, four subunits of the S TAGA complex, and Mot1. TAFs that are also present in S TAGA are indicated by asterisks. Bottom: schematic diagrams of the promoter constructs are shown. (B) Transcription analysis in ts13 cells, which harbor a temperature-sensitive mutation in TAF1. Ts13 cells grown at the permissive or non-permissive temperature were transiently transfected with a luciferase reporter plasmid and a plasmid expressing a transcriptional activator. Transcription was monitored by luciferase activity, and normalized relative to activity at the permissive temperature. (C) Left: immunoblot analysis is shown. 293A cells were transfected with a TAF5 or TAF12 shRNA expression vector or an empty vector (control) and analyzed by immunoblotting using the indicated antibodies. Right: transcription levels were monitored by luciferase reporter activity in shRNA-treated cells, and normalized relative to luciferase activity in non-shRNA-treated cells.

To investigate further the composition of the TC formed on the HIV-1 LTR, we obtained a panel of antibodies directed against nine additional human TAFs (TAF2, TAF4, TAF6, TAF7, TAF8, TAF9, TAF11, TAF12, and TAF13), as well as antibodies against four subunits of the S TAGA complex (PAF65β, hSpt3, hGCN5, and TRRAP) and the TBP-interacting protein Mot1. The results (Figure 3A) show that when transcription was directed by Gal4-VP16, all 11 TAFs analyzed were bound to the promoter, as expected for recruitment of TFIID. By contrast, when transcription was directed by Tat, none of the 11 TAFs were recruited to the HIV-1 LTR. Gal4-VP16 also recruited all four S TAGA subunits analyzed, none of which were associated with the HIV-1 LTR in the presence of Tat. Finally, Mot1 was not recruited when transcription was directed by either Gal4-VP16 or Tat. On the basis of these data we conclude that the TC formed on the HIV-1 LTR lacks at least 11, and probably all, of the 14 TFIID TAFs. In the experiments presented below, TAF1 and TAF5 were analyzed as representative TAFs.

### TAFs Are Not Required for Tat-Mediated Transcription Activation

The results of the ChIP experiments strongly suggested that transcription activation by Tat did not require TAFs. To confirm this supposition, we examined the ability of Tat to stimulate transcription following TAF inactivation. First, we examined the ability of Tat to activate transcription in ts13 cells, which harbor a temperature-sensitive mutation in TAF1 [29,30]. Figure 3B shows, as expected, that inactivation of TAF1 significantly diminished transcription activation by VP16 and E1a, whereas Tat-mediated transcription activation was unaffected. In a second approach, we used short hairpin RNAs (shRNAs) to knockdown expression of TAF5 or TAF12 in 293A cells. Immunoblot analysis confirmed that shRNA-mediated knockdown of TAF expression was efficient and specific (Figure 3C, left). Transcriptional analysis revealed that knockdown of TAF5 or TAF12 substantially decreased transcription directed by VP16, whereas Tat-mediated transcription activation was unaffected (Figure 3C, right). (Because 293A cells are transformed by and express E1a, activation by E1a could not be analyzed in this experiment.) Collectively, the results of Figure 3 demonstrate that TAFs are not involved in transcription activation directed by the HIV-1 Tat protein.

### Tat and P-TEFb Direct Recruitment of TBP in the Absence of TAFs

The results described above indicate that on the HIV-1 LTR transcription activation involves assembly of an atypical TC that contains TBP but not TAFs. However, these experiments do not distinguish whether the critical determinant for recruitment of TBP and not TFIID is Tat or the promoter, the HIV-1 LTR. To address this issue we performed a series of artificial tethering experiments.

**Figure 4:**
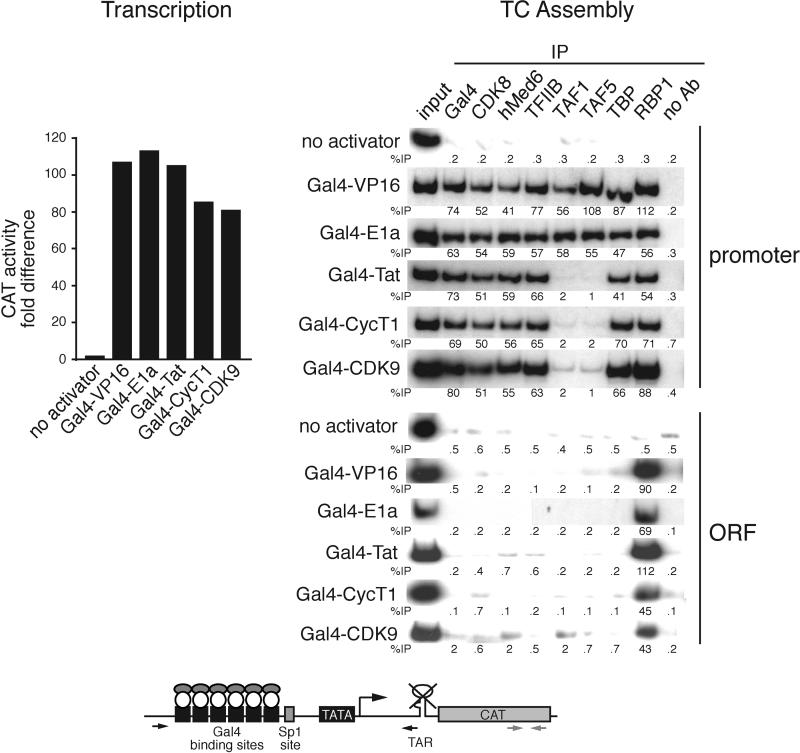
Tat and P-TEFb Direct Recruitment of TBP in the Absence of TAFs When Tethered to DNA Transcription (left) and TC assembly (right) were examined using an HIV-1 LTR derivative that lacks the TAR element and contains upstream Gal4-binding sites (G6[−83]HIV LTRΔTARCAT; bottom) and a series of Gal4 fusion proteins, as indicated.

Previous studies have shown that Tat can activate transcription when directed to the HIV-1 LTR through a heterologous DNA-binding domain [31,32,33]. We analyzed TC assembly using an HIV-1 LTR derivative that lacked the TAR element and contained upstream Gal4-binding sites. We first examined the TCs formed on this promoter by three Gal4 fusion proteins: Gal4-VP16, Gal4-E1a, and Gal4-Tat. Consistent with previous studies [32], Figure 4 shows that all three Gal4 fusion proteins activated transcription from this modified HIV-1 LTR derivative. The accompanying ChIP experiments show that Gal4-VP16 and Gal4-E1a supported assembly of a TC that contained all of the GTFs, including TAF1 and TAF5. By contrast, Gal4-Tat directed assembly of a TC in which the TAFs were present at a level significantly below that of TBP and other GTFs. These results indicate that the activator Tat, and not the HIV-1 LTR promoter, directs the selective recruitment of TBP in the absence of TAFs.

As discussed above, Tat interacts directly with CycT1, a subunit of the transcription elongation factor P-TEFb. Thus, Tat is an adaptor whose function is to recruit P-TEFb. We therefore considered that P-TEFb was ultimately responsible for the selective recruitment of TBP in the absence of TAFs. To address this possibility, we analyzed transcription activation by the two P-TEFb subunits, CycT1 and CDK9. Consistent with previous results [34,35], both Gal4-CycT1 and Gal4-CDK9 activated transcription. The ChIP analysis indicates that both Gal4-CycT1 and Gal4-CDK9 stimulated assembly of a TC that contained all of the GTFs but lacked TAF1 and TAF5. These results confirm that P-TEFb is responsible for directing selective recruitment of TBP in the absence of TAFs.

In the experiments shown in Figure 4, P-TEFb was tethered to DNA, whereas on the HIV-1 LTR, P-TEFb is normally bound through Tat to nascent RNA. We therefore sought to verify that P-TEFb, when bound to nascent RNA, would also selectively recruit TBP in the absence of TAFs. Previous studies have shown that recruitment of CycT1/P-TEFb to the HIV-1 LTR through a heterologous RNA-binding domain can activate transcription in the absence of Tat [36]. We therefore asked whether such an artificially recruited CycT1/P-TEFb protein would stimulate assembly of a TC that contained TBP but not TAFs. We used a previously characterized HIV-1 LTR derivative [36] in which the TAR element was replaced by a minimal binding site for the HIV-1 Rev protein, and examined the ability of a Rev-Tat or Rev-CycT1 fusion protein to stimulate TC assembly.

**Figure 5:**
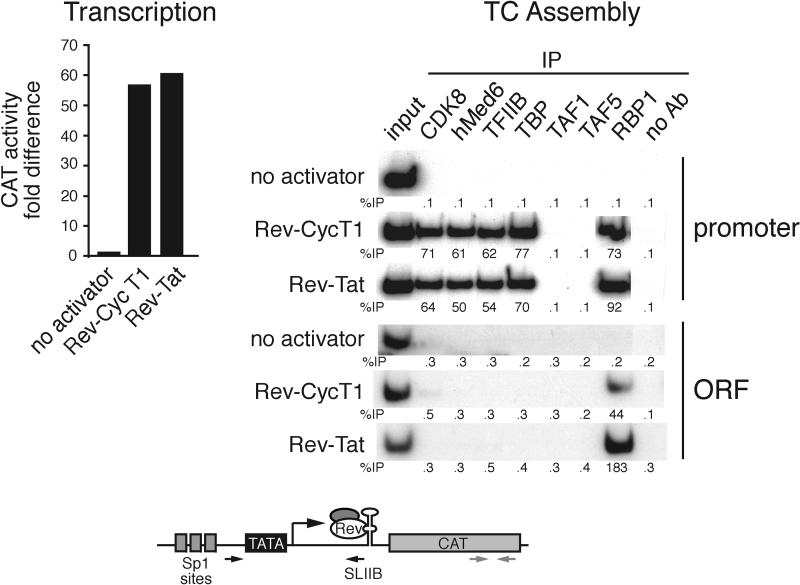
Tat and P-TEFb Direct Recruitment of TBP in the Absence of TAFs When Tethered to RNA Transcription (left) and TC assembly (right) were examined using a Rev-Tat or Rev-CycT1 fusion protein and an HIV-1 LTR derivative in which the TAR element was replaced by stem loop IIB (SLIIB), a Rev-protein-binding site (HIV SLIIBCAT; bottom).

Figure 5 shows that in the absence of a Rev fusion protein, both transcription and GTF recruitment were virtually undetectable. Addition of Rev-Tat or Rev-CycT1 resulted in a large transcriptional increase, as expected from previous studies. Significantly, addition of Rev-Tat or Rev-CycT1 also led to a large increase in recruitment of TBP, TFIIB, mediator, and RNA polymerase II, but not TAF1 or TAF5. Thus, in the absence of Tat, Rev-CycT1 stimulated assembly of a TC that contained TBP but not TAFs.

### Identification of Cellular Promoters That Recruit TBP in the Absence of TAFs

P-TEFb is also a cofactor for several cellular activators, the best studied of which is CIITA, a transcription factor involved in the expression of major histocompatibility complex (MHC) class II genes (reviewed in [37]). In fact, overexpression of Tat can inhibit transcription of MHC class II genes by competing with CIITA for P-TEFb binding [38,39].

**Figure 6:**
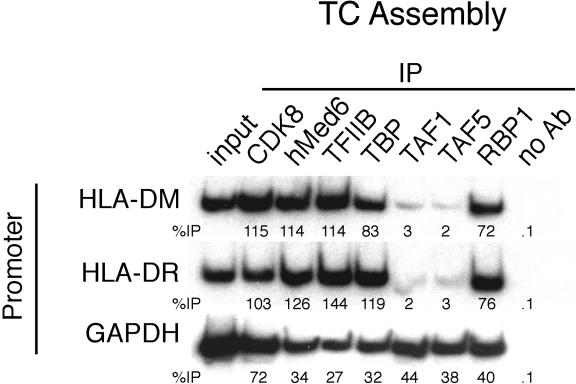
The Cellular Activator CIITA Directs Recruitment of TBP in the Absence of TAFs TC assembly was monitored on the promoters of *HLA-DM* and *HLA-DR,* two MHC class II genes known to use CIITA, and, as a negative control, *GAPDH*.

We therefore tested whether the promoters of MHC class II genes also recruited an atypical TC that contained TBP but not TAFs. We analyzed two MHC class II genes known to use CIITA, *HLA-DM* and *HLA-DR,* and, as a negative control, *GAPDH*. RT-PCR analysis confirmed, as expected, that both *HLA-DM* and *HLA-DR* were expressed in 293T cells (data not shown). The associated ChIP experiment (Figure 6) shows that the TCs formed on both *HLA-DM* and *HLA-DR* contained all of the GTFs but lacked significant levels of TAF1 and TAF5. By contrast, the TC formed on *GAPDH* contained all of the GTFs, including TAF1 and TAF5. These results indicate that TBP, and not TFIID, is also selectively recruited to specific cellular promoters and strongly support the conclusion that P-TEFb directs this recruitment.

## Discussion

A variety of studies have proposed that Tat promotes transcriptional elongation [11,12,13]. However, the effect of Tat on TC assembly in vivo has not been previously analyzed. In this report, we studied the mechanism of Tat action using ChIP assays and found, unexpectedly, that Tat dramatically stimulates TC assembly on the HIV-1 LTR. Perhaps most surprising, the HIV-1 LTR contains an atypical TC that lacks TFIID and that has not been previously described in mammalian cells. By contrast, transcription activation by Gal4-VP16, the SV40 enhancer, or the Ad E1a protein involves assembly of a TC that contains TFIID. Thus, our results reveal mechanistic similarities between apparently diverse classes of activators and unanticipated differences in the composition of mammalian TCs.

In an HIV-1-infected cell, a low level of Tat-independent transcription can be elicited by cellular activators, such as Sp1, NF-κB, and NFAT, which are bound to upstream regions of the HIV-1 LTR [12]. Tat is recruited to the vicinity of the promoter by binding to this low level of nascent TAR RNA, where it facilitates subsequent rounds of TC assembly and transcription initiation. Thus, once a threshold level of transcription is achieved, Tat stimulates additional transcription, thereby increasing the number of Tat-binding sites (reviewed in [40]). This positive feedback mechanism would ensure that viral transcription rapidly increases or decreases, which may be relevant to the ability of HIV-1 to enter latency and, conversely, to be activated from the latent state.

### Tat Stimulates TC Assembly

Previous studies have provided two principal lines of evidence that Tat functions by stimulating transcription elongation. First, Tat has been found to stimulate elongation in an in vivo nuclear run-off assay [41,42,43,44], and in the absence of Tat, apparent prematurely terminated transcripts have been detected [41,45]. However, these in vivo results are complicated by the presence of a second promoter, the initiator of short transcripts, adjacent to the HIV-1 LTR [46,47]. In our experiments RNA polymerase II was not detected either near or far downstream of the transcription start site in the absence of Tat and thus provided no evidence for a paused (or stalled) RNA polymerase II. Consistent with our ChIP data, nuclear run-off experiments have shown that Tat increases the density of RNA polymerase II 9- to 15-fold within the first 25 nucleotides downstream of the transcription start site [42,43], indicating that Tat also stimulates initiation. A second line of evidence has been derived from in vitro experiments in which Tat was found to enhance elongation but not initiation (see, for example, [48]). However, in vitro transcription experiments may fail to faithfully recapitulate in vivo regulation: for example, under in vitro conditions using naked DNA templates, PIC assembly and initiation may no longer be rate-limiting on the HIV-1 LTR. Consistent with this possibility, the HIV-1 LTR is a relatively strong promoter when analyzed as naked DNA in vitro in the absence of Tat (see, for example, [49]). Moreover, initiation effects would not be observed if the cell-free system did not support multiple rounds of transcription from a single DNA template (see, for example, [50]).

It is important to emphasize that our ChIP assay only analyzed TC assembly. Thus, our results do not rule out the possibility that in addition to stimulating TC assembly, Tat also promotes transcription elongation. Based upon the results presented here and previous studies (reviewed in [11,12,13]), we believe that Tat stimulates TC assembly, thereby promoting initiation and elongation. This dual mechanism of action may explain why Tat is such a potent activator of transcription.

### Tat and P-TEFb Direct Recruitment of TBP in the Absence of TAFs

We have shown that transcription from the HIV-1 LTR involves TBP but not TFIID. This result was particularly unexpected because in mammalian cells TBP is bound to TAFs extremely tightly and numerous biochemical studies have failed to find free TBP [2,3,4]. None of the 11 TAFs, four S TAGA subunits, or Mot1 was present on the HIV-1 LTR, strongly suggesting that free TBP is recruited. However, we cannot exclude the possibility that TBP may be associated with unknown proteins that remain to be identified.

Several considerations rule out the possibility that TFIID is actually present in the TC directed by Tat/P-TEFb but that the TAFs are not detected in the ChIP assay. First, numerous studies have shown ChIP to be a remarkably general and robust assay that has successfully detected a wide variety of activators, general initiation and elongation components, chromatin remodeling factors, and histone variants. Second, in experiments not involving Tat or P-TEFb, TAFs were detected at levels comparable to that of TBP on five different promoters (G4E1bCAT, *β-globin*, E4CAT, G6(−83)HIV LTRΔTARCAT, and *GAPDH*). Third, when TC assembly was directed by Tat or P-TEFb the ChIP assay detected all GTFs analyzed, including TBP, but not TAFs on six different promoters ([−83]HIV LTRCAT, integrated HIV-1/LAV, HIV SLIIBCAT, G6[−83]HIV LTRΔTARCAT, *HLA-DM*, and *HLA-DR*]. Finally, and perhaps most persuasively, on the same promoter the presence or absence of TAFs as monitored by ChIP was dictated solely by the upstream bound Gal4 fusion protein (see Figure 4).

Artificial tethering experiments clearly demonstrate that P-TEFb directs recruitment of TBP in the absence of TAFs. Strongly supporting this conclusion is our identification of cellular promoters for which P-TEFb is a cofactor and whose TC contains TBP but not TAFs. How P-TEFb selectively recruits TBP and not TFIID remains to be determined. It has been previously thought that P-TEFb is purely an elongation factor [14,15]. However, we have shown that when tethered to DNA or nascent RNA, P-TEFb can also stimulate TC assembly and dictate the composition of the TC.

In yeast, promoters have been grouped into two extreme classes based on their requirement for TAFs [16,17]. TAF-dependent promoters require TAFs for transcription, and on these promoters TBP and TAFs are present at comparable levels. TAF-independent promoters do not require TAFs for activity, and on these promoters TAFs are either absent or present at levels far below that of TBP. These yeast results are strikingly similar to the findings reported here, and taken together these results indicate that in both yeast and mammalian cells, transcription of protein-coding genes involves alternative TCs that differ by the presence or absence of TAFs.

## Materials and Methods

### 

#### Plasmids

To generate the G4E1bCAT construct, pDSG39, four Gal4-binding sites were inserted upstream of the E1b TATA box and the CAT ORF in the vector pSP72 (Promega, Madison, Wisconsin, United States). The *β-globin* constructs containing (pBS) or lacking (pB/E) the SV40 enhancer were provided by Walter Schaffner; pBS contains a 196-bp SV40 enhancer–containing fragment inserted into a plasmid derived from pB6 [51], which harbors the *β-globin* gene. The E4CAT construct, pE4CAT, was previously described [52]. The pFR-Luc plasmid, containing five Gal4-binding sites upstream of the E1b TATA box and the luciferase reporter gene, was obtained from Stratagene (La Jolla, California, United States). The HIV LTR-luciferase construct was previously described [53]. The (−83)HIV LTRCAT and G6(−83)HIV LTRΔTARCAT constructs, were previously described [54]. The HIV SLIIBCAT construct, pHIV/SLIIB/CAT [36], was provided by Bryan Cullen.

Plasmids expressing the Gal4-VP16 fusion protein (pGal4-VP16; [55]), E1a (pSV-E1a; [52]), Tat (CMV-Tat; [56]), and the Gal4-E1a fusion protein (pGal4-E1a; [52]) were previously described. To generate pGal4-Tat, pGal4-CycT1, and pGal4-CDK9, the full-length Tat protein, the hCycT1-containing EcoR1 fragment from phCycT1-Rev [36], or the CDK9-containing EcoR1 fragment from pSG5-CDK9 (a gift from Claude Gazin), respectively, were individually cloned into plasmid pSG424 [55] as an in-frame fusion with the Gal4 DNA-binding domain. Rev fusion-protein plasmids pTAT-Rev and phCycT1-Rev [36] were obtained from Bryan Cullen.

#### Antibodies

The α-Gal4 mouse monoclonal (sc-510), α-CDK8 goat polyclonal (sc-1521), α-CDK9 goat polyclonal (sc-7331), α-CycT1 goat polyclonal (sc-8127), α-Med6 goat polyclonal (sc-9434), α-GCN5 goat polyclonal (sc-6303); α-TRRAP rabbit polyclonal (sc-11411), α-TAF1 mouse monoclonal (sc-735), α-TAF5 mouse monoclonal (sc-743), α-Sp1 rabbit polyclonal (sc-59X), and α-alpha tubulin mouse monoclonal (sc-5268) antibodies were obtained from Santa Cruz Biotechnology (Santa Cruz, California, United States). The α-RNA polymerase II mouse monoclonal antibody (8WG16) was obtained from BAbCO (Berkeley, California, United States). The α-Tat rabbit antiserum was obtained from the National Institutes of Health AIDS Research and Reference Reagent Program (catalog number 705; [57]). The α-TBP mouse monoclonal antibody (SL30–3-563) was provided by Nouria Hernandez; the α-TAF2, α-TAF4, α-TAF6, α-TAF7, α-TAF8, α-TAF9, α-TAF11, α-TAF12, α-TAF13, and α-hSpt3 rabbit polyclonal antibodies were provided by Robert Roeder; the α-Mot1 rabbit polyclonal antibody was provided by Franklin Pugh; and the α-PAF65β rabbit polyclonal antibody was provided by Yoshihiro Nakatani.

#### Transcription

Transcription was measured either by RT-PCR analysis from total RNA isolated from transfected cells or by chloramphenicol acetyl transferase (CAT) assay [58] using ^14^C chloramphenicol followed by thin layer chromatography. Signals were visualized by autoradiography and quantitated using National Institutes of Health Image 1.62 software.

#### ChIP assay

HeLa and 293T cells were transfected with 10 μg of effector plasmids and 5 μg of reporter plasmid using standard CaCl_2_ transfection methods [59]. Forty-eight hours after transfection, cells were cross-linked by adding formaldehyde to the medium (1% final concentration) and incubated for 10 min at room temperature. The cross-linking reaction was quenched by adding glycine to a final concentration of 0.125 M and incubating for an additional 10 min at room temperature. Cells were then washed with ice-cold PBS containing protease inhibitors, scraped in the same buffer, and washed in PBS. Cells were then lysed in SDS-lysis buffer (1% SDS, 50 mM Tris-HCl [pH 8.0], 10 mM EDTA, and protease inhibitors), and sonicated at least 6–8 times at output 8 for 10 s, with 2 min incubation on ice in between. Lysates were centrifuged at 15,000 rpm for 15 min at 4 °C; 5% of the lysates were kept as input. Lysates were diluted 10-fold with ChIP dilution buffer (0.01% SDS, 1.1% Triton X-100, 1.2 mM EDTA, 16.7 mM Tris-HCl [pH 8.0], 16.7 mM NaCl, and protease inhibitors) and pre-cleared with 80 μl of Protein A Agarose-50% Slurry (Upstate Biotechnology, Lake Placid, New York, United States) for 30 min at 4 °C with constant agitation. The agarose beads were pelleted, and the supernatant was incubated overnight with the primary antibody. To each tube, 50 μl of Salmon Sperm DNA/Protein A Agarose-50% Slurry (Upstate Biotechnology) was added, and the mixture was incubated for 1 h at 4 °C. The agarose beads were pelleted by gentle centrifugation, the supernatant was removed, and the pellet was washed twice with low-salt immune complex wash buffer (0.1% SDS, 1% Triton X-100, 2 mM EDTA, 20 mM Tris-HCl [pH 8.0], and 150 mM NaCl), once with high-salt immune complex wash buffer (0.1% SDS, 1% Triton X-100, 2 mM EDTA, 20 mM Tris-HCl [pH 8.0], and 500 mM NaCl), once with LiCl immune complex wash buffer (0.25 M LiCl, 1% NP40, 1% C_24_H_39_NaO_4_, 1 mM EDTA, and 10 mM Tris-HCl [pH 8.0]), and twice with TE (10 mM Tris-HCl [pH 8.0] and 1 mM EDTA). Chromatin was eluted with freshly prepared elution buffer (1% SDS and 0.1 M CHNaO_3_). After reversal of the cross-links, the samples were treated with proteinase K for 1 h at 45 °C, extracted by phenol/chloroform, and ethanol precipitated. The pellet was resuspended in TE, and PCR was performed. Autoradiograms were scanned and quantitated by the National Institutes of Health ImageJ program. IP DNA was quantitated and presented as the ratio of IP to input. Primer sequences are available upon request.

#### TAF inactivation

For TAF1 inactivation, ts13 cells [60] were initially cultured at the permissive temperature (33 °C), after which one set of 6-well plates was shifted to the non-permissive temperature (39.5 °C) while another set was kept at 33 °C. Sixteen hours later, cells were co-transfected with a plasmid expressing a transcriptional activator (Gal4-VP16, E1a, or Tat) and a luciferase reporter plasmid, and incubated further for 24 h prior to measuring transcription by luciferase assay. For shRNA-mediated inactivation of TAFs, 293A cells were transfected with either pcDNA3 (Invitrogen, Carlsbad, California, United States) or a TAF5 or TAF12 shRNA expression vector (Open Biosystems, Huntsville, Alabama, United States), and 18 h later were co-transfected with effector and reporter plasmids. Transcription activation was monitored by luciferase activity 24 h following transfection.

## Supporting Information

### Accession Numbers

The LocusLink (http://www.ncbi.nlm.nih.gov/LocusLink/) accession numbers for the genes and gene products discussed in this paper are CDK8 (1024), CDK9 (1025), CIITA (4261), CycT1 (904), *GAPDH* (2597), hGCN5 (2648), *HLA-DM* (3109), *HLA-DR* (3122), hSpt3 (8464), MED6 (10001), Mot1 (9044), PAF65β (27097), RBP1 (5430), TAF1 (6872), TAF11 (6882), TAF12 (6883), TAF13 (6884), TAF2 (6873), TAF4 (6874), TAF5 (6877), TAF6 (6878), TAF7 (6979), TAF8 (129685), TAF9 (6880), Tat (155871), TBP (6908), TFIIB (2959), and TRRAP (8295).

## Abbreviations

Ad: adenovirus
ChIP: chromatin immunoprecipitation
CycT1: cyclin T1
GTF: general transcription factor
HIV-1: human immunodeficiency virus type I
LTR: long terminal repeat
MHC: major histocompatibility complex
ORF: open reading frame
PIC: pre-initiation complex
P-TEFb: positive transcription elongation factor b
shRNA: short hairpin RNA
TAF: TATA-box-binding protein associated factor
TBP: TATA-box-binding protein
TC: transcription complex
